# Ten‐year work burden after prostate cancer treatment

**DOI:** 10.1002/cam4.6530

**Published:** 2023-09-19

**Authors:** Samuel L. Washington, Peter E. Lonergan, Janet E. Cowan, Shoujun Zhao, Jeanette M. Broering, Nynikka R. Palmer, Cameron Hicks, Matthew R. Cooperberg, Peter R. Carroll

**Affiliations:** ^1^ Department of Urology, Helen Diller Family Comprehensive Cancer Center University of California San Francisco California USA; ^2^ Department of Epidemiology & Biostatistics University of California San Francisco California USA; ^3^ Department of Urology St. James's Hospital Dublin Ireland; ^4^ Department of Surgery, School of Medicine Trinity College Dublin Dublin Ireland; ^5^ Department of Surgery University of California San Francisco California USA; ^6^ Department of Medicine University of California San Francisco California USA; ^7^ Division of General Internal Medicine Zuckerberg San Francisco General Hospital San Francisco California USA

**Keywords:** disease management, prostate cancer, racial groups, work burden

## Abstract

**Introduction:**

We aim to characterize the magnitude of the work burden (weeks off from work) associated with prostate cancer (PCa) treatment over a 10‐year period after PCa diagnosis and identify those at greatest risk.

**Materials and Methods:**

We identified men diagnosed with PCa treated with radical prostatectomy, radiation therapy, or active surveillance/watchful waiting within CaPSURE. Patients self‐reported work burden and SF36 general health scores via surveys before and 1,3,5, and 10 years after treatment. Using multivariate repeated measures generalized estimating equation modeling we examined the association between primary treatment with risk of any work weeks lost due to care.

**Results:**

In total, 6693 men were included. The majority were White (81%, 5% Black, and 14% Other) with CAPRA low‐ (60%) or intermediate‐risk (32%) disease and underwent surgery (62%) compared to 29% radiation and 9% active surveillance. Compared to other treatments, surgical patients were more likely to report greater than 7 days off work in the first year, with relatively less time off over time. Black men (RR 0.64, 95% CI 0.54–0.77) and those undergoing radiation (vs. surgery, RR 0.46, 95% CI 0.41–0.51) were less likely to report time off from work over time. Mean baseline GH score (73 [SD 18]) was similar between race and treatment groups, and stable over time.

**Conclusions:**

The work burden of cancer care continued up to 10 years after treatment and varied across racial groups and primary treatment groups, highlighting the multifactorial nature of this issue and the call to leverage greater resources for those at greatest risk.

## Graphical abstract

Within this longitudinal study of 6693 men treated for prostate cancer, time take off from work due to PCa treatment persisted up to 10 years after primary treatment. The long‐term impact of PCa treatment was not limited only to those with paid jobs and was found to also effect one's usual activities/responsibilities and general health of all men with PCa.

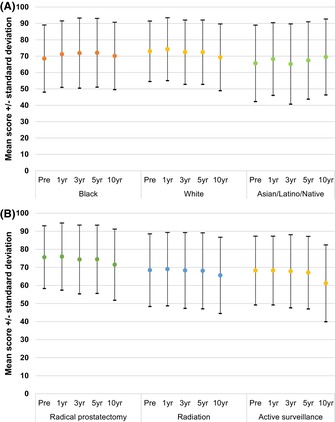

## 1 INTRODUCTION

Approximately 288,300 men will be diagnosed with prostate cancer (PCa) in 2023 in the US.^1^ For men newly diagnosed with PCa, selection of the most appropriate treatment option, such as radical prostatectomy (RP), radiation therapy (external beam radiation therapy or brachytherapy (RT)), or active surveillance/watchful waiting (AS/WW) depends upon clinical risk.^2^, ^3^, ^4^ Each treatment modality requires consideration of various factors including age, comorbidities, and tumor characteristics in order to optimize outcomes for patients, yet factors such as employment, life responsibilities, and one's ability to take time off for cancer care remain excluded from standardized risk assessments used to inform treatment decision.^5^, ^6^, ^7^ Regardless of the modality used, undergoing treatment and post‐treatment follow‐up will require that time spent at work or managing other responsibilities will now be devoted to their cancer care which may include clinic visits, lab testing, and additional imaging tests.^8^, ^9^ This work burden remains a concern for patients that may not be routinely addressed by providers and is not addressed within treatment guidelines.^2^, ^3^, ^4^, ^10^

The work burden of cancer treatment is not limited to the treatment period itself; cancer surveillance after treatment routinely lasts for at least 5–10 years with the frequency of clinic visits and testing changing over time.^2^ As a result, the work burden of treatment and one's ability to attend clinic visits remains dynamic; it varies by the type of treatment received and the frequency of post‐treatment surveillance.^8^ These dynamic factors may be influenced by an individual's social risk characteristics (e.g., education, income, access to health services, greater financial hardship, inability to leave work for care) and affect their ability to take time away from work for cancer care.^11^, ^12^, ^13^ Prior European studies have examined aspects of work burden although restricted to a single treatment modality such as RP or excluded those not currently employed.^8^, ^14^ As a result, little data can be generalized to men from more vulnerable populations within the United States (e.g., Black men, those who are unemployed, or of lower socioeconomic status) who may face a greater work burden.

Little is known of the magnitude or duration of work burden after treatment for PCa. We hypothesized that work burden decreases but persists over time, after primary treatment. Using a longitudinal, observational study of primarily community‐based urology practices within the Cancer of the Prostate Strategic Urologic Research Endeavor (CaPSURE) database, we aim to characterize the magnitude of the work burden associated with PCa management over a 10‐year period after PCa diagnosis and identify those at greatest risk. A greater understanding of this work burden of cancer treatment can aid in identifying those at greatest need of supportive resources and inform more comprehensive shared decision‐making discussions of treatment and survivorship for men newly diagnosed with PCa.

## 2 METHODS

### 2.1 Overview

**FIGURE 1:**
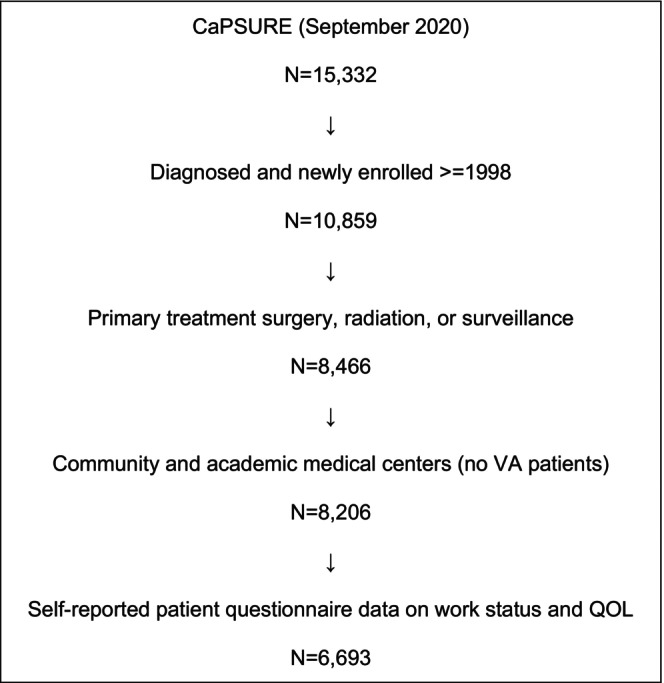
CONSORT (Consolidated Standards of Reporting Trials) diagram for creation of study cohort.

Participants were enrolled in CaPSURE, a longitudinal, observational registry of 15,332 men with all stages of biopsy‐proven PCa at 43 community urology practices, academic medical centers, and Veteran's hospitals throughout the United States. The CaPSURE study focus was solely on clinical and treatment data from inception in 1995 through 1998, after which the scope was expanded to include patient‐reported questionnaires to determine comorbid conditions, symptoms, medication use, employment details, health care resource utilization, and quality of life. Men newly diagnosed and enrolled after 1998 who underwent primary management with RP, RT, or AS/WW within 6 months after first positive biopsy were included (Figure 1). Men who predated the questionnaire period, had incomplete primary treatment data, or lacked reported data on work status were excluded. Patients completed surveys at regular time points: at diagnosis and at 1, 3, 5, and 10 years after treatment. This cohort study was conducted with approval of the institutional review boards at the University of California, San Francisco and at all participating CaPSURE sites (10‐00881, study 95982). Written informed consent was obtained from all study participants.

Demographic, clinicopathologic, and follow‐up clinical and laboratory data were abstracted from the CaPSURE database. Both patients and their physicians reported race and ethnicity (Black, White, Asian/Pacific Islander, Latino, Native American/Alaskan/Hawaiian, mixed). Each patient reported his relationship status (partnered, single), education level (some high school, high school degree, some college, college degree), annual household income (<$30,000, $30,000‐50,000, $50,000‐75,000, >$75,000), and insurance coverage (Medicare, Medicare with supplemental insurance, private, Veterans Health Administration (VA) funded) at time of initial evaluation. Patients reported health related behaviors at diagnosis such as current smoker, alcohol intake (7+ drinks per week), and body mass index (BMI) (<25, 25 to <30, ≥30) and completed a checklist for previous medical history based on the Charlson comorbidity index at diagnosis. Year of diagnosis, serum PSA at diagnosis, and clinical T stage were abstracted from the database. Biopsy Gleason Grade Group (GG) and percentage of biopsy cores positive were obtained from the pathology records. Preoperative risk stratification was calculated using the Cancer of the Prostate Risk Assessment Score (CAPRA).^6^ Clinical site of care was categorized as academic, community, or veterans as established within the CaPSURE framework, and US census regions were based on site location. Primary treatment type was collected and grouped by RP, radiotherapy (BT, EBRT), and AS/WW.

### 2.2 Survey responses about health care, employment, and quality of life

PCa visits included PSA testing, imaging and diagnostics, inpatient stays, and outpatient visits with a urologist, radiation oncologist, or medical oncologist. Patients reported type of employment or career at diagnosis and updated job status on subsequent surveys (see Data S1). Job titles were classified according to the Standard Occupational Classification from 2018.^15^ Occupations were further categorized as administrative/non‐physical versus physical labor. At each timepoint men reported whether time off from “work” (i.e., job or usual/home activities/responsibilities) was required to visit a physician, psychologist, or other healthcare professional for each individual health care visit. Number of work weeks lost, defined as missing >7 days of work due to PCa care, was computed from the patient's responses to four employment questions: the number of hours cut or limited from work or usual activity, the number of days completely unable to carry out work or usual activities, and the number of days spent in bed for more than half a day because of poor health, appointments, or related care. Although time off from paid work would reflect a 5‐day week, a 7‐day “work” week was selected to reflect home/unpaid responsibilities not bound by the five‐day paid work week. *General health* was quantified using the 36‐Item Short Form Survey (SF‐36) administered at each pre‐specified time point as a measure of overall quality of life and health, as this could affect time spent away from work.

### 2.3 Statistical analysis

Summary statistics for demographic and clinical characteristics at the time of presentation were generated. Mean with standard deviation (SD) and median with interquartile range (IQR) were utilized to describe continuous variables. Frequency tables and proportions were calculated for categorical variables. Differences by primary treatment type and by race were assessed using *t*‐test, Kruskal–Wallis test and chi‐squared statistic.

In preparation for multivariable analysis, a separate category represented missing values for each independent variable with incomplete data; no patient records were excluded for statistical analysis due to missing data. Time ranges were used for dependent variables to maximize all available employment and SF‐36 data; for example, the 1‐year time point utilized data up to 365 days after treatment or start of AS/WW and the 3‐year time point utilized data from 366 to 1095 days after treatment. Follow‐up interval was calculated from time of treatment to last contact.

The multivariable analysis tested the association between primary treatment, adjusted for independent covariables, with risk of the outcome of interest, *any work weeks lost due to care (yes/no)*, at 1, 3, 5, and 10 years after treatment. The statistical method was repeated measures generalized estimating equation (GEE) modeling, a regression model designed to estimate the likelihood of longitudinal data, such as any work weeks lost, assuming correlations between patient responses over time. A series of preliminary unadjusted models was performed to test each independent variable described above.

The final set of model covariates, based on preliminary findings and a priori decisions, included characteristics at diagnosis (age, clinical CAPRA risk score,^6^ number of comorbid conditions, current smoker, work status, job type (physical, non‐physical)), race and ethnicity, US census region, type of clinical site, and type of primary treatment. Race/ethnicity was aggregated as Black, White, and other (Asian/Pacific Islander, Latino, Native American/Alaskan/Hawaiian, mixed) due to distribution of values. Work status was grouped into two categories: paid work (full‐time, part‐time) and unpaid responsibilities (caregiver, volunteer, retired, student, on leave/unemployed, disabled, unspecified). The GEE model included primary treatment as a random effect with a time interaction term. This approach allowed for assessment of within‐ and between‐treatment group variation of loss of work over time. The remaining covariates were included as fixed effects. A two‐sided *p* < 0.05 was considered significant. Analysis was performed with SAS 9.4 for Windows.

## 3 RESULTS

### 3.1 Cohort characteristics

Among 15,332 men ever enrolled in CaPSURE, 8206 men were diagnosed in 1998–2017 at community‐based or academic medical centers and primarily managed with surgery, radiation, or surveillance (Figure 1). Most had CAPRA low‐ (0–2, 60%) or intermediate‐risk (3–5, 32%) disease. 62% underwent RP, 29% RT, and 9% AS/WW (Table 1). Of men diagnosed, 6693 (82%) reported work status and formed the analytic cohort: (5% Black; 81% White; 14% Asian, Latino, or Other) with a mean age of 64 years (SD 8.3) (Table 1). Most were married (91%) and had at least some college education (64%). Few smoked (9%) while most endorsed alcohol intake (≥7 per week, 64%). Most described their jobs as administrative or non‐physical (78%) and made less than $75,000 per year (67%). Nearly half reported having a paid job (33% full time, 12% part‐time). Nearly all were insured (57% private, 30% Medicare + supplemental, 13% Medicare). Median follow‐up was 6.7 years (IQR 3.6–11.7). Primary treatment types were RP (62%), brachytherapy (14%), external beam radiation (15%), and AS/WW (9%). The excluded group of patients (*n* = 1513), due to missing patient‐reported questionnaire data, had similar age and clinical characteristics at diagnosis, but underwent more non‐surgical primary treatments (48% vs. 38%) and had shorter post‐treatment follow‐up (median 3 vs. 7 years), both *p* < 0.01.

**TABLE 1:** Demographic and clinical characteristics at diagnosis of study cohort, overall and stratified by race/ethnicity and by primary treatment.

Patient characteristics	Value	All patients		Black		White		Asian, Latino, Native			RP		RAD		AS/WW		
		Med	IQR	Med	IQR	Med	IQR	Med	IQR	*p*	Med	IQR	Med	IQR	Med	IQR	*p*
Age	Years	65	58, 70	62	56, 67	65	59, 70	64	57, 69	<0.01	62	56, 66	70	64, 74	71	65, 77	<0.01
Comorbidities	No. per year	2	1, 3	2	1, 3	2	1, 3	2	1, 3	0.72	1	1, 2	2	1, 3	2	1, 3	<0.01
		*N*	(%)	*N*	(%)	*N*	(%)	*N*	(%)	*p*	*N*	(%)	*N*	(%)	*N*	(%)	*p*
Race/ethnicity	Native	12	0					12	6	–	12	0	0	0	0	0	<0.01
	Asian/Pacific	40	1					40	21		21	1	11	1	8	1	
	Latino	87	1					87	46		39	1	38	2	10	2	
	Black	358	5	358	80						213	5	108	6	37	6	
	White	5402	81			5402	89				3339	81	1544	80	519	82	
	Mixed	27	0					27	14		19	0	6	0	2	0	
	Unknown	767	11	92	20	651	11	24	13		484	12	229	12	54	9	
Education	Some HS	641	11	111	31	489	9	41	25	<0.01	275	8	287	17	79	14	<0.01
	HS degree	1474	25	87	25	1350	25	37	23		851	23	487	29	136	24	
	Some college	1164	20	67	19	1065	20	32	20		747	21	301	18	116	20	
	College degree	2616	44	90	25	2474	46	52	32		1766	49	608	36	242	42	
	Missing	798		95		675		28			488		253		57		
Income	<$30,000	1503	26	157	47	1283	25	63	40	<0.01	654	19	672	41	177	33	<0.01
	30–50,000	1205	21	78	23	1098	21	29	18		718	20	380	23	107	20	
	50–75,000	1118	20	48	14	1050	20	20	13		766	22	261	16	91	17	
	>75,000	1873	33	52	16	1776	34	45	29		1388	39	316	19	169	31	
	Missing	994		115		846		33			601		307		86		
Smoker	No	5364	91	293	83	4925	92	146	89	<0.01	3291	90	1538	91	535	93	0.12
	Yes	534	9	62	17	454	8	18	11		346	10	148	9	40	7	
	Missing	795		95		674		26			490		250		55		
Alcohol drinks	<7 per week	2086	36	183	52	1826	34	77	48	<0.01	1229	34	642	38	215	38	<0.01
	7+	3785	64	166	48	3534	66	85	52		2398	66	1034	62	353	62	
	Missing	822		101		693		28			500		260		62		
Clinical site	Academic	634	9	27	6	564	9	43	23	<0.01	491	12	80	4	63	10	<0.01
	Community	6059	91	423	94	5489	91	147	77		3636	88	1856	96	567	90	
US Region	West	873	13	34	8	784	13	55	29	<0.01	549	13	185	10	139	22	<0.01
	East	2819	42	245	54	2502	41	72	38		1691	41	909	47	219	35	
	Midwest	2203	33	119	26	2047	34	37	19		1552	38	423	22	228	36	
	South	798	12	52	12	720	12	26	14		335	8	419	22	44	7	
Job status	Unpaid	3677	55	263	58	3308	55	106	56	0.03	1892	46	1319	68	466	74	<0.01
	FT paid	2223	33	152	34	2003	33	68	36		1771	43	355	18	97	15	
	PT paid	793	12	35	8	742	12	16	8		464	11	262	14	67	11	
Job type	Admin	5220	78	334	74	4747	78	139	73	0.03	3231	78	1496	77	493	78	0.66
	Physical	1473	22	116	26	1306	22	51	27		896	22	440	23	137	22	
CAPRA risk	Low 0–2	3501	60	193	54	3221	60	87	53	0.02	2214	61	886	53	401	75	<0.01
	Intermed 3–5	1871	32	126	35	1680	32	65	40		1183	32	574	34	114	21	
	High 6–10	476	8	38	11	426	8	12	7		252	7	206	12	18	3	
	Missing	845		93		726		26			478		270		97		
Treatment	RP	4127	62	272	60	3747	62	108	57	<0.01	4127	62					–
	BT	937	14	48	11	855	14	34	18				937	14			
	EBRT	999	15	91	20	883	15	25	13				999	15			
	AS/WW	630	9	39	9	568	9	23	12						630	9	

Abbreviations: AS/WW, active surveillance/watchful waiting; BT, brachytherapy; CAPRA, clinical Cancer of the Prostate Clinical Risk Assessment; EBRT, external beam radiation; FT, full time employment; HS, high school; IQR, interquartile range; Med, median; Native, Native American/Alaskan/Hawaiian; P, Kruskal–Wallis *p*‐value for comparison of median values (age only) or chi‐square *p*‐values for comparison of categorical values.; PT, part time employment; RAD, radiotherapy; RP, radical prostatectomy.

### 3.2 Frequency of visits, imaging, and labs

Study participants reported a median of 2.3 PSA tests (IQR 1, 17.4), 5.6 imaging studies (IQR 1.8, 19.7), and 1.3 (IQR 1, 1.7) clinic visits related to PCa care per year (Table 2). Black men were noted to have a greater number of PCa related evaluations per year (median 5.7 [IQR 1.8, 17.7], 3.7 [IQR 1.6, 13.2] for White men, 5.0 [IQR 2, 14.2] for Asian/Latino/Native American men, <0.01), largely driven by an increased frequency of imaging studies per year (8.1 [IQR 2.6, 24.1] for Black men, 5.4 [IQR 1.8, 19.2] for White men, 6 [IQR 2.4, 21.9] for Asian/Latino/Native American men, *p* < 0.01). By treatment, radiotherapy patients had a greater number of PCa related evaluations per year (median 4.9 [IQR 2.4, 13] vs. 3.7 [IQR 1.5, 16.6] for surgical patients and 2.1 [1.3, 3.7] for men on AS/WW, <0.01).

**TABLE 2:** Prostate cancer related visits of the study cohort, overall and stratified by race/ethnicity and by primary treatment.

Prostate cancer visits	All patients		Black		White		Asian, Latino, Native			RP		RAD		AS/WW		
Prostate cancer visits	*N*	Median	*N*	Median	*N*	Median	*N*	Median	*p*	*N*	Median	*N*	Median	*N*	Median	*p*
PSA tests per year	119	2.3	9	16.6	104	2.3	6	2.2	0.62	55	1.8	52	2.6	12	1.8	0.20
Imaging tests per year	4677	5.6	312	8.1	4230	5.4	135	6.0	<0.01	2706	7.8	1593	4.6	378	1.7	<0.01
Visits per year	5729	1.3	318	1.3	5271	1.3	140	1.2	0.55	3610	1.2	1636	1.3	483	1.1	<0.01
All evaluations per year	6409	3.8	406	5.7	5831	3.7	172	5.0	<0.01	3971	3.7	1872	4.9	566	2.1	<0.01

Abbreviations: AS/WW, active surveillance/watchful waiting; BT, brachytherapy; EBRT, external beam radiation; IQR, interquartile range; Med, median (per patient); Native, Native American/Alaskan/Hawaiian; P, Kruskal–Wallis *p*‐value for comparison of median values; PSA, prostate specific antigen; RAD, radiotherapy; RP, radical prostatectomy.

### 3.3 Weeks off from work over time

**FIGURE 2:**
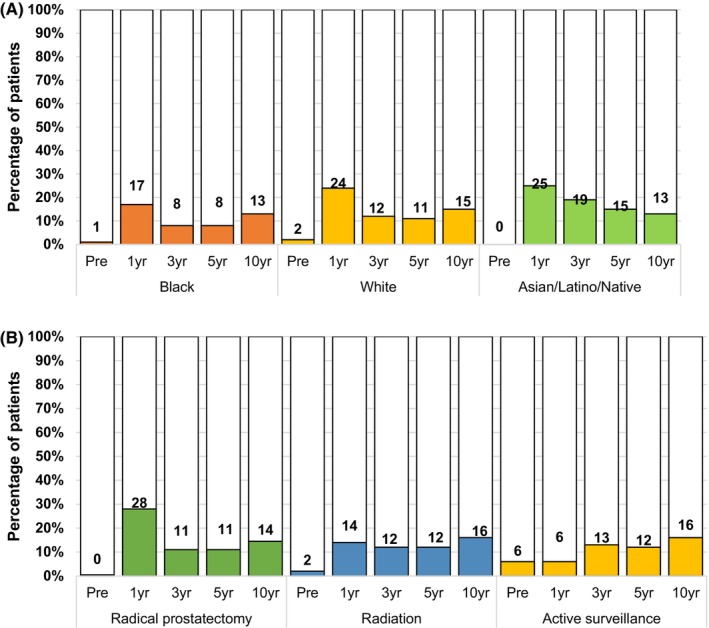
Proportion of patients who missed >7 days of work due to prostate cancer care (A) by race and (B) by treatment. Missed days (yes/no) defined as any patient‐reported work days that were limited, missed, or spent in bed.

The proportion of men who reported time off from work (in weeks) due to cancer care ranged from 12% prior to treatment up to 15% at 10 years post‐treatment. A lower percentage of Black men reported time off from work at all timepoints compared to White and Asian/Latino/Native American counterparts (Figure 2A). More men who underwent RP reported time off from work compared to RT and AS/WW in the first‐year post‐treatment with a subsequent decrease over time (Figure 2B).

On multivariable analysis, Black men (RR 0.64, 95% CI 0.54–0.77), older age (per 10‐year increase, RR 0.84, 95% CI 0.79–0.88), and being treated at a community center (RR 0.85, 95% CI 0.77–0.94) were associated with lower risk of taking time off work at various time points after PCa treatment (Table 3). Higher clinical risk at diagnosis (by CAPRA, vs. low, RR 1.21, 95% CI 1.06–1.37) and greater comorbidity burden (1–2 vs. none, RR 1.19, 95% CI 1.08–1.29; 3–4, RR 1.68, 95% CI 1.52–1.86; ≥5, RR 2.23, 95% CI 1.89–2.63) were associated with greater risk of taking time off work at various time points after PCa treatment. Those who underwent RT had a lower risk of needing time off from work over time (RR 0.46, 95% CI 0.41–0.51, Table 3).

**TABLE 3:** Long‐term changes in reported time off work after primary treatment for prostate cancer (in weeks) using multivariable repeated measures generalized estimating equation (GEE) modeling, with treatment*time interaction term.

Parameter	Category	RR (95% CI)	*p*_parameter_	*p*_global_
Race/ethnicity	Black vs. non‐Black	0.64 (0.54–0.77)	<0.01	<0.01
Age	Per 10 years	0.84 (0.79–0.88)	<0.01	<0.01
Clinical CAPRA	High 6–10 vs. low 0–2	1.21 (1.06–1.37)	<0.01	0.03
	Intermediate 3–5 vs. low 0–2	1.06 (0.99–1.14)	0.10	
	Missing vs. low 0–2	1.01 (0.91–1.11)	0.89	
Comorbidities	1–2 vs. none	1.19 (1.08–1.29)	<0.01	<0.01
	3–4 vs. none	1.68 (1.52–1.86)	<0.01	
	≥5 vs. none	2.23 (1.89–2.63)	<0.01	
	Missing vs. none	1.07 (0.85–1.35)	0.55	
Work status	Full time paid vs. unpaid	1.06 (0.99–1.15)	0.11	0.07
	Part time paid vs. unpaid	1.11 (1.01–1.22)	0.03	
Job type	Labor/physical vs. admin/non‐physical	0.95 (0.88–1.03)	0.22	0.21
US region	East vs. West	1.03 (0.93–1.14)	0.58	<0.01
	Midwest vs. West	0.91 (0.82–1.00)	0.06	
	South vs. West	1.04 (0.91–1.20)	0.55	
Type of clinical site	Community vs. academic	0.85 (0.77–0.94)	<0.01	<0.01
Current smoker	Missing vs. no	1.34 (1.07–1.68)	<0.01	0.03
	Yes vs. no	0.95 (0.84–1.08)	0.45	
Primary treatment	AS/WW vs. radical prostatectomy	1.00 (1.00–1.00)		<0.01
	Radiotherapy vs. radical prostatectomy	0.46 (0.41–0.51)	<0.01	
Time	3 years vs. 1 year	0.32 (0.29–0.34)	<0.01	<0.01
	5 years vs. 1 year	0.33 (0.30–0.36)	<0.01	
	10 years vs. 1 year	0.40 (0.37–0.43)	<0.01	
Primary treatment*time	3‐year AS/WW	1.16 (0.92–1.46)	0.20	<0.01
	5‐year AS/WW	1.36 (1.05–1.77)	0.02	
	10‐year AS/WW	1.22 (0.95–1.56)	0.12	
	3‐year radiotherapy	2.42 (2.10–2.79)	<0.01	
	5‐year radiotherapy	2.39 (2.03–2.82)	<0.01	
	10‐year radiotherapy	2.36 (2.02–2.75)	<0.01	

Abbreviations: AS/WW, active surveillance/watchful waiting; CAPRA, clinical cancer of the prostate risk assessment; CI, confidence interval; RR, relative risk.

### 3.4 Changes in general health

**FIGURE 3:**
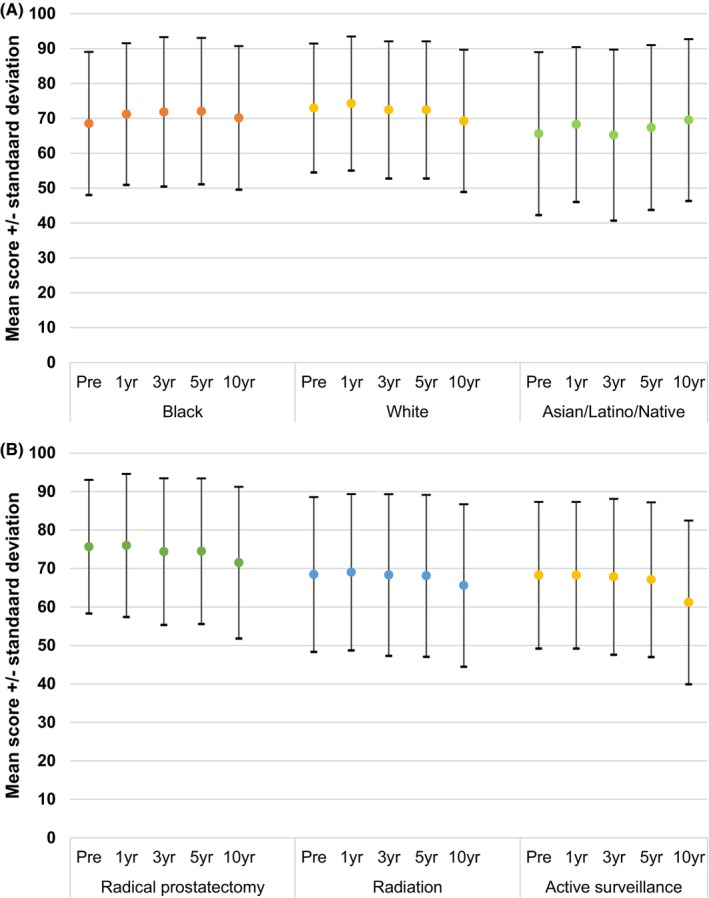
Unadjusted mean SF36 General Health scores before and after primary treatment of the study cohort, stratified by (A) race/ethnicity and (B) primary treatment.

In terms of general health, mean baseline score was 73 (SD 18) and did not vary significantly between race groups or treatment groups. Post‐treatment trends in general health remained stable over time (Figure 3).

## 4 DISCUSSION

Within this longitudinal study of 6693 men treated for PCa, up to 15% of men endorsed a persistent work burden of more than 7 days off from work or usual activities due to PCa treatment up to 10 years after primary treatment. Despite a greater number of PCa‐related evaluations over time, Black men were less likely to report taking time off from work at all timepoints (RR 0.64, 95% CI 0.54–0.77). Job status (full‐time paid vs. unpaid work) and job type (labor/physical vs. administrative/non‐physical) were not associated with long‐term risk of work burden, even after adjustment for treatment, and follow‐up duration.

Monetary costs (out‐of‐pocket or insurance‐related) form the basis of discussions surrounding financial toxicity of cancer treatment as they are more readily quantifiable, yet non‐monetary sequelae of treatment such as work burden may also contribute to overall financial hardship and influence care. The time lost from work or usual activities due to cancer treatment represents an “indirect cost” that contributes to the overall financial toxicity experienced by patients receiving treatment.^11^ Prior work has explored the costs of the PCa diagnostic pathway,^16^, ^17^, ^18^, ^19^, ^20^ from screening to treatment but less is known about indirect costs such as work burden. European studies have explored sick leave after primary treatment of localized PCa and work status after surgical treatment of PCa for working men^8^, ^14^; our study is the largest diverse cohort with long‐term follow‐up to characterize work burden in men undergoing PCa treatment, irrespective of employment status. By including a diverse cohort with long‐term follow‐up after treatment that includes employed and unemployed or retired men, our findings provide more generalizable and broader insight into the potential hardship of treatment and post‐treatment follow‐up on those undergoing treatment with the United States. Prior studies have shown that financial hardship of cancer treatment may lead to increased anxiety independent of changes in the actual financial burden,^21^ and decreases in care utilization which further impair quality of life.^12^ One study of cancer survivors of breast, lung, colorectal, or PCa found that those experiencing hardships such as decreased income were 4.4 (95% CI 2.9–6.6) times more likely to not attend follow‐up treatment and encounters.^12^ Similarly, others found that patients disclosing financial toxicity were more likely to report delays in care due to the inability to take time off work for cancer care and afford general expenses.^22^ From a treatment standpoint, those who underwent RP reported greater work burden during the first year but harbored less risk over time. Those managed with AS/WW reported low but persistent work burden over time, likely reflecting the need for repeated assessments of PCa status (labs and imaging) whereas those treated with surgery or RT usually are followed with periodic assessment of PSA alone. A Swedish study examining the duration of sick leave in 15,902 working men after AS, RP, or RT found that men choosing AS reported fewer days of sick leave within the first 5 years after diagnosis compared to other treatments (17 days vs. 46 days for RP vs. 44 days for RT).^8^ In this study the authors note there were no differences by treatment at 5 years. With longer follow‐up and without restricting only to working men, our study demonstrates that work burden not only persists up to 10 years but also varies by treatment type. These differences may be attributable to the longer reported follow‐up, differences in sociodemographics, and broader inclusion criteria to capture the impact of care on work and usual activities. These findings can help patients understand to what degree they can anticipate needing for recovery and post‐treatment cancer surveillance over time. For those with limited ability to take time off of work, this information can better inform treatment decisions and post‐treatment expectations long‐term.

Within this study Black men reported lower income, higher risk disease at diagnosis, and were 36% less likely to report time off from work despite an increased frequency of imaging compared to other groups (8.1 [IQR 2.6, 24.1] for Black men, 5.4 [IQR 1.8, 19.2] for White men, 6 [IQR 2.4, 21.9] for Asian/Latino/Native American men, *p* < 0.01). These observations bring into question whether the decreased work burden over time signifies an inability to take time off due to socioeconomic factors and an unmeasured financial burden. The CEASAR study explored this burden further in 2359 men with clinically localized PCa through surveys related to financial toxicity and treatment regret.^23^ The authors found that 4.3% of men at 3‐year follow‐up and 3.6% of men at 5‐year follow‐up endorsed a large or very large financial burden; these men were more likely to be non‐white, unemployed, and have greater disease burden. The overall financial burden was associated with greater treatment regret. These findings are particularly concerning given the disproportionate burden of disparities in PCa that Black men face^24^ and prior work illustrating the relationship between financial hardship, lower income, and the inability to leave work for care.^22^ Prior work using patient surveys in men with metastatic PCa reported that older age, applying for assistance programs, and an annual income >$100,000 were associated with lower financial hardship; those with greater hardship needed to limit spending on basic goods, recreational activities, and use saving in order to pay for treatment.^25^ The confluence of these social and financial factors may in turn result in disruptions in cancer treatment, recovery, and follow‐up for Black men with greater social needs, thereby contributing to known racial disparities in PCa outcomes. These findings further support the need for efforts to understand patient perspectives to enrich our understanding of the impact of these social needs on their care as they progress through the care pathway. Yet integrating decisions of treatment costs into the shared decision‐making process has had limited benefit for patients,^26^ suggesting that focusing on costs alone may ignore other social needs impacting decisions and care. Efforts to obtain patient‐level perspectives do not obviate the role that healthcare organizations must play in integrating screening for these factors into the clinical encounter, understanding the prevalence of these risk factors within their own catchment area, and exploring interventions to connect social resources to the patients they serve.

Limitations are inherent in this analysis. This observational study relied upon self‐reported time off from work due to PCa treatment, thereby introducing potential recall or participation bias from participants. This study was not initially designed to examine drivers of these observations. Lastly, 6% of the cohort self‐identified as Black, which represents an area of underrepresentation that remains consistent in the literature and limits generalizability to the broader US population. Despite these limitations, the strengths of this study include this being the largest national cohort of men within the United States to date to characterize the magnitude of burden resulting due to PCa treatment. In addition, the cohort still represents the largest national cohort of men overall, and Black men specifically (*n* = 466), to examine work burden, particularly for men treated in community urology practices. By leveraging specific questions regarding the impact of cancer care on work and usual activities over time, this study represents the first study to our knowledge to present data on changes in work burden both longitudinally and at the person‐level. As a result, this study offers a novel view of how treatment impacts the lives of men with PCa treatment over time that has not yet been reported to this degree within the literature. Based on this work, prospective collection of social risk factors data within our patient population has commenced in hopes of early identification of those at greater risk, more personalized counseling of treatment options and post‐treatment expectations of work burden, and tailor interventions linking patients to social resources that may mitigate the work burden over time.

## 5 CONCLUSIONS

Nearly one‐quarter of men with PCa reported a persistent work burden due to PCa management up to 10 years after treatment. This work burden was associated with clinical characteristics, race, age, and clinical site, highlighting its multifactorial nature of and the need for prospective assessments to identify those at greatest risk.

## AUTHOR CONTRIBUTIONS

**Samuel L. Washington:** Conceptualization (lead); formal analysis (equal); methodology (supporting); project administration (supporting); writing – original draft (lead); writing – review and editing (equal). **Peter E. Lonergan:** Conceptualization (equal); investigation (equal); writing – review and editing (equal). **Janet E. Cowan:** Conceptualization (equal); formal analysis (lead); methodology (equal); software (lead); visualization (equal); writing – review and editing (equal). **Shoujun Zhao:** Data curation (equal); formal analysis (equal); methodology (equal); visualization (equal); writing – review and editing (equal). **Jeanette M. Broering:** Data curation (equal); investigation (equal); methodology (equal); project administration (equal); writing – review and editing (equal). **Nynikka R. Palmer:** Conceptualization (equal); investigation (equal); supervision (equal); writing – review and editing (equal). **Cameron Hicks:** Data curation (equal); methodology (equal); visualization (equal); writing – review and editing (equal). **Matthew R. Cooperberg:** Conceptualization (equal); methodology (equal); project administration (equal); supervision (equal); visualization (equal); writing – review and editing (equal). **Peter R. Carroll:** Funding acquisition (equal); project administration (equal); supervision (equal); writing – review and editing (equal).

## FUNDING INFORMATION

UCSF Goldberg‐Benioff Program in Translational Cancer Biology Conflicts of interest: none.

## Supporting information


Data S1.

## Data Availability

Due to its proprietary nature and ethical concerns, supporting data cannot be made openly available. Further information about the data and conditions for access are available upon request.
