# Supplementary material for: Ten‐year work burden after prostate cancer treatment
Source: Cancer Med. 2023 Sep 19;12(18):19234–44. doi: 10.1002/cam4.6530 (PMC10557888; doi:10.1002/cam4.6530)
Supplement: Supplementary file 1 — Data S1. [file CAM4-12-19234-s001.docx]

**Supplemental Materials**

**Employment questions for CaPSURE patient-reported questionnaire administered before primary treatment and at yearly intervals after primary treatment.**

| 1) **Which of the following choices best describes your current work status?** |
| --- |
| Responses: “work full-time at any job”, “work part-time because of your health”, work part-time unrelated to your health”, “on leave from work”, “temporarily laid-off”, “unemployed or looking for work”, “disabled and unable to work”, “retired”, and “other”. |
|  |
| 2) **From January 1 – December 31,2017, have you taken any time off from work to visit your doctor, a psychologist, or other health professionals?** |
| Responses: “No” or “Yes” with listing of hours |
|  |
| 3) **From January 1 – December 31,2017, how many days have you had to cut down or limit your work or usual activities because of your health?** |
| Responses: write in 0 or number of days |
|  |
| 4) **From January 1 – December 31,2017, how many days have you been COMPLETELY UNABLE TO CARRY OUT your work or usual activities BECAUSE OF YOUR HEALTH?** |
| Responses: write in 0 or number of days |
|  |
| 5) **From January 1 – December 31,2017, how many days did you stay in bed more than half a day BECAUSE OF YOUR HEALTH?** |
| Responses: write in 0 or number of days |

**Response rates for CaPSURE patient-reported questionnaire and study status at last patient contact.**

| Patient-reported questionnaire response rate | Value | N | (%) |
| --- | --- | --- | --- |
| Questionnaire completed pre-treatment | No | 3601 | 44 |
|  | Yes | 4605 | 56 |
| Questionnaire completed at 1 year | No | 541 | 10 |
|  | Yes | 4874 | 90 |
|  | Missing | 2791 | . |
| Questionnaire completed at 3 years | No | 90 | 2 |
|  | Yes | 4882 | 98 |
|  | Missing | 3234 | . |
| Questionnaire completed at 5 years | No | 52 | 1 |
|  | Yes | 3598 | 99 |
|  | Missing | 4556 | . |
| Questionnaire completed at 10 years | No | 31 | 1 |
|  | Yes | 2760 | 99 |
|  | Missing | 5415 | . |
| Study status | Withdrew | 2227 | 27 |
|  | Clinical only | 2403 | 29 |
|  | Lost to follow up | 172 | 2 |
|  | Deceased | 1573 | 19 |
|  | Full participation | 1376 | 17 |
|  | Questionnaire only | 455 | 6 |
